# The Association Between Serum Vitamin D Status and Cardiometabolic Risk Factors in Extremely Obese Adults: A Cross-Sectional Study

**DOI:** 10.1155/ije/7945302

**Published:** 2025-06-26

**Authors:** Yan Zhou, Chunyan Wang, Zhixing Wang, Xiaojing Wang

**Affiliations:** ^1^Department of Endocrinology, Aviation General Hospital, Beijing, China; ^2^Department of Endocrinology, Beijing Tsinghua Changgung Hospital, Tsinghua University, Beijing, China

**Keywords:** lipids, obesity, vitamin D deficiency

## Abstract

**Background:** Vitamin D may play a vital role in glucose and lipid metabolism, but existing studies on this topic are limited and inconsistent. The aim of our study was to undertake an in depth exploration of the relationship between vitamin D and cardiometabolic markers in extremely obese individuals in North China.

**Methods:** In this cross-sectional study, 293 adults aged between 18 and 60 years with BMIs higher than 35 kg/m^2^ were recruited from Beijing, China. Serum 25(OH) D was measured using chemiluminescence, and concentrations of glucose, insulin, and lipids were quantified as well. Multiple linear regression analysis adjusted for potential confounding factors was used to assess independent associations between serum 25(OH) D and lipid profiles.

**Results:** The prevalence of vitamin D deficiency was 81.2% in the extremely obese subjects, and the median serum 25(OH) D concentration was 12.88 ng/mL. Moreover, 25(OH) D levels were significantly negatively associated with the BMI and parathyroid hormone (*p* < 0.0001). After adjustment for age, sex, BMI, and HOMA-IR, 25(OH) D levels were positively correlated with HDL-c (*p* < 0.007) and ApoA1 as well (*p* < 0.001). No significant differences in blood pressure, fasting blood glucose, or HOMA-IR were detected between vitamin D nondeficient (≥ 20 ng/mL) and deficient (< 20 ng/mL) groups.

**Conclusions:** Vitamin D deficiency is highly prevalent in extremely obese individuals in China, exhibiting significant correlations with higher BMI and lower HDL-c/ApoA1. Our findings underscore the importance of vitamin D deficiency as a correlate of cardiovascular risk. Prospective studies and randomized clinical trials are warranted to determine the effect of 25(OH) D on other cardiometabolic parameters.

## 1. Introduction

Obesity has become a global epidemic that poses a tremendous challenge to healthcare management [1]. China currently bears the world's highest obesity burden, with its prevalence rate experiencing a fivefold increase since 2004. This epidemiological shift correlates strongly with urbanization, sedentary lifestyles, and dietary transitions towards energy-dense foods [2]. Obesity increases the risk of major chronic diseases, such as type 2 diabetes [3], certain cancers [4], and cardiovascular disease (CVD) [5], which is the leading cause of morbidity and mortality globally. Moreover, an inverse association between vitamin D and obesity has been frequently observed, and increased vitamin D deficiency in obese patients has been reported as well [6]. Obesity-related vitamin D deficiency may stem from vitamin D sequestration in adipose tissue and insufficient cutaneous vitamin D synthesis due to diminished outdoor exposure.

25-hydroxyvitamin D [25(OH) D] is well known for its central role in calcium and phosphorous homeostasis and bone metabolism. In recent years, emerging evidence suggests that vitamin D has various extra-skeletal effects, particularly its roles in cardiomyocytes, vascular endothelium, vascular smooth muscles, and pancreatic beta-cells. [7, 8]. Many observational studies have reported that vitamin D deficiency is a major global health problem linked to CVD and cardiometabolic disease risk factors (obesity, hyperglycemia, hypertension, and dyslipidemia) [7, 9]. The interplay between vitamin D and obesity further exacerbates the effects of vitamin D deficiency on CVD development in patients with obesity. Nevertheless, the reported relationships between the vitamin D status and traditional CVD risk factors remain inconsistent [10]. Furthermore, one recent meta-analysis that included more than 83,000 individuals showed that vitamin D supplementation was not associated with reduced major adverse cardiovascular events [11]. To date, although substantial studies have investigated the association between the vitamin D status and cardiometabolic parameters in obese and overweight populations, evidence remains limited in individuals with extreme obesity-a subgroup at higher metabolic risk. Additionally, the relationship has not been adequately studied in specific ethnic populations and in Chinese adults in particular. Thus, in the present study, we aimed to evaluate whether serum vitamin D is related to cardiometabolic risk factors in extremely obese adults from North China.

## 2. Materials and Methods

### 2.1. Subjects

The subjects in this study were enrolled from the hospital between January, 2020 and December, 2021. The inclusion criteria were as follows: age between 18 and 60 years and BMI higher than 35 kg/m^2^, irrespective of concurrent type 2 diabetes mellitus, hypertension, or metabolic syndrome. The exclusion criteria were as follows: genetic or endocrine causes of obesity, severe liver or renal diseases, lipid-lowering drug use, taking vitamin D or calcium or mineral supplements in the last 3 months prior to blood extraction, and unavailable serum vitamin D data. This study was approved by the local Ethics Committee of our hospital (no. 24303-6-01).

### 2.2. Clinical and Laboratory Assessment

A trained nurse measured blood pressure, body height, body weight, and waist circumference (WC) according to standard protocols. Venous blood samples for biochemical measurements were drawn after overnight fasting. Liver and renal function parameters and serum lipid profiles were measured using an automatic biochemical analyzer (Roche C702, Germany). Glycosylated hemoglobin (HbA1c) was determined using high-performance liquid chromatography (BIO-Rad VARIANT II), and serum insulin and C-peptide levels were measured using the direct chemiluminescence immunoassay (Roche E801, Germany). Serum 25(OH) D and parathyroid hormone (PTH) were quantified by a chemiluminescence method (YaHuiLong iFlash 3000, China) and an electrochemiluminescence method (Roche E801, Germany), respectively. The intra-assay and interassay coefficients of variation of the 25(OH) D assay were 8% and 10%, respectively. HOMA-IR was used to evaluate basal insulin resistance. The vitamin D status was categorized based on serum 25(OH) D concentrations: deficiency (< 20 ng/mL), insufficiency (20–29 ng/mL), and sufficiency (> 30 ng/mL) [12].

### 2.3. Statistical Analysis

Continuous variables were expressed as mean ± standard deviation or median and interquartile range depending on their distributions. The Kolmogorov–Smirnov test was used to assess whether these variables were normally distributed. Differences in clinical and metabolic parameters among subjects stratified by gender and vitamin D status were compared using Student's *t*-test or the Mann–Whitney *U*-test. Categorical variables were expressed as counts (percentages) and were analyzed using the chi-squared test. Associations between serum 25(OH) D levels and metabolic parameters were evaluated using Spearman correlation. Multiple linear regression analysis adjusted for potential confounding factors was used to examine the independent effect of serum 25(OH) D on lipid profiles. Two models were estimated. In the first model, age, sex, and BMI were adjusted, and in the second model, HOMA-IR was adjusted additionally. A *p* value < 0.05 was the threshold for statistical significance for all tests, and all statistical analyses were performed using SPSS version 20.0.

## 3. Results

### 3.1. General Characteristics of the Subjects

A total of 293 extremely obese subjects were studied, and 75.8% of these were female. The study population had a mean age of 36.61 ± 7.42 years and a mean BMI of 42.04 ± 6.10 kg/m^2^. The median serum 25(OH) D concentration was 12.88 ng/mL. Female subjects exhibited a lower BMI, WC, uric acid, and creatinine levels compared with males. Higher serum PTH and lower 25(OH) D levels were also observed in females. Additionally, female subjects had higher low-density lipoprotein cholesterol (LDL-c), high-density lipoprotein cholesterol (HDL-c), and apolipoprotein A1 (ApoA1) than males. The general characteristics of the subjects stratified by sex are shown in Table 1.

### 3.2. Metabolic Phenotypes Classified by Serum 25(OH) D

Vitamin D deficiency was found in 81.2% of these extremely obese subjects. Furthermore, vitamin D insufficiency and vitamin D sufficiency were present in the remaining 18.8% of patients, who were classified together as the vitamin D nondeficient group. Compared to vitamin D nondeficient participants, vitamin D deficient participants were older and had a higher BMI and LDL-c, as well as lower HDL-c and ApoA1, but the difference in LDL-c was not statistically significant. Furthermore, we found that PTH levels were significantly higher in vitamin D deficiency subjects. However, no significant differences were found when comparing blood pressure, fasting blood glucose, fasting insulin, or HOMA-IR between the two groups.  Table 2 shows the metabolic phenotypes between different vitamin D statuses.

### 3.3. The Relationship Between Serum 25(OH) D and Metabolic Parameters

**Figure 1:**
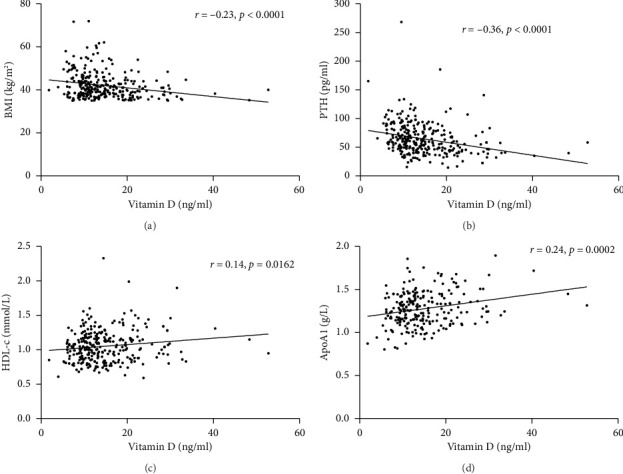
Association between 25(OH) D and BMI, PTH, HDL-c, and ApoA1.

Spearman correlation analysis showed that total vitamin D was negatively associated with the BMI (*r* = −0.23, *p* < 0.0001) and PTH (*r* = −0.36, *p* < 0.0001) but was positively correlated with HDL-c (*r* = 0.14, *p*=0.0162) and ApoA1 (*r* = 0.24, *p*=0.0002) (Figure 1). In-line, multiple linear regression analysis indicated that a higher 25(OH) D level (crude analysis) was associated with higher HDL-c and ApoA1 (Table 3) as well. When adjusting for the confounders, age, sex, and BMI (model 1), the association between 25(OH) D and HDL-c and ApoA1 not only persisted but also became stronger. Finally, after additionally adjusting for HOMA-IR, the above-mentioned associations still remained significant (Model 2).

## 4. Discussion

Our study revealed an alarmingly high prevalence of vitamin D deficiency in a cohort of extremely obese adults from North China. Our findings showed that individuals with vitamin D deficiency exhibited a higher BMI and PTH and that serum 25(OH) D concentrations were significantly negatively related to the BMI and PTH. Furthermore, our study demonstrated that there was a strong positive association between 25(OH) D and HDL-c, even after adjusting for several confounders. We also found a positive association between serum 25(OH) D and ApoA1. However, no significant differences in other cardiometabolic risk factors (blood pressure, fasting glucose, fasting insulin, and HOMA-IR) between vitamin D nondeficient (≥ 20 ng/mL) and deficient (< 20 ng/mL) patients were observed.

High burden of vitamin D deficiency was observed in our study population, with fewer than 20% of subjects exhibiting serum 25(OH) D levels above the adequacy threshold of 20 ng/mL. Indeed, the poor vitamin D status has been reported previously in elderly Chinese individuals in Beijing, where 69.2% of participants showed 25(OH) D levels ≤ 50 nmol/L [13]. Similar to our findings, the Peking Vertebral Fracture study showed that the 25(OH) D levels of postmenopausal women in Beijing were remarkably low, with an average of only 13.2 ± 5.4 ng/mL [14]. Additionally, a study of adolescent girls in Beijing suggested that more than 40% of school-aged girls had 25(OH) D levels below 12.5 nmol/L [15]. These data demonstrate that vitamin D deficiency is common in both children and adults in Beijing, China.

In our cohort, several factors likely contributed to the high prevalence of the low vitamin D status, such as the relatively high latitude of Beijing (latitude 40° north), the popular use of umbrellas, hats, and sunscreens when outdoors, lack of physical activity due to obesity, and limited availability of vitamin D supplements or fortified foods in supermarkets. Therefore, we recommend routine vitamin D supplementation for individuals with obesity in North China.

Numerous observational studies have shown that vitamin D deficiency is positively associated with the BMI [16–18]. Evidence from a 45-year-old British birth cohort involving 7198 Caucasian subjects confirmed the inverse relationship between vitamin D and obesity [19]. A meta-analysis further substantiated the significant negative correlation between the vitamin D status and the BMI [20]. Moreover, researchers have reported that vitamin D is negatively associated with perirenal fat thickness - a validated indicator of visceral adiposity [21]. Cheng et al. similarly suggested that both subcutaneous adiposity and visceral adiposity are inversely related to vitamin D levels [22]. Consistent with previous studies, our results revealed that a decrease in 25(OH) D levels was associated with an increased BMI in extremely obese individuals. What is more, a meta-analysis involving 3153 participants reported that vitamin D supplementation had a beneficial and significant reduction in the BMI in the subgroups of Asian with an intervention duration of more than 6 months [23]. Current perspectives propose that increased adipose tissue volumes in obese people sequester fat-soluble vitamin D, decrease vitamin D bioavailability, and reduce the release of vitamin D into the circulation [24, 25]. Additionally, obese individuals usually have less outdoor physical activity and inadequate sun exposure, leading to reduced synthesis of vitamin D in the skin [26].

Current evidence also suggests that vitamin D plays a role in lipoprotein metabolism. Notably, our results indicated that a poor vitamin D status was associated with lower HDL-c levels independent of the BMI, age, sex, and HOMA-IR. This finding was concordant with other vitamin D and lipid association studies, which showed that higher vitamin D levels were associated with increasing serum cardioprotective HDL-c levels [27–29]. In one study of 7952 adults with overweight or obese, vitamin D deficiency was positively correlated with reduced HDL-c levels [30]. More specifically, a cross-sectional study explored the association between HDL particle composition and vitamin D levels in postmenopausal women and demonstrated that higher 25(OH) D levels were associated with the large HDL particle subclass, which plays a vital role in reverse cholesterol transport and contributes to athero-protective function [31]. Additionally, a large meta-analysis that assessed the pooled effect of vitamin D supplementation on lipids in 39 randomized controlled trials (RCTs) indicated that vitamin D supplementation significantly increased HDL-c [32]. However, another meta-analysis reported inconsistent results and found no significant effects on HDL-c after vitamin D supplementation in prediabetic individuals [33]. A recent systematic review also showed that vitamin D supplementation did not yield significant alterations in HDL-c in individuals with obesity or overweight [34]. In addition, a Serial Clinical Laboratory Data Study of more than 100,000 patients suggested that correcting for a vitamin D deficiency might not translate into clinically meaningful improvement in lipid profiles, although higher 25(OH) D levels were associated with higher HDL-c [35]. Therefore, rigorous RCTs are needed to clarify the causal relationship between the vitamin D status and cardiometabolic markers.

ApoA1, an important functional component of HDL-c, is associated with reduced CVD risk. The cardioprotective effects of ApoA1 are primarily mediated through facilitating reverse cholesterol transport and anti-inflammatory actions. Additionally, emerging evidence demonstrates that ApoA1 exerts regulatory effects on energy homeostasis in adipocytes. ApoA1-deficient mice exhibited greater body weight gain and adipose tissue accumulation relative to WT controls [36]. Obese individuals are characterized by reduced HDL-c and ApoA1 levels [30]. In the present study, we found that there was a positive relationship between serum 25(OH) D and ApoA1 levels after adjustment for potential confounders. This is in line with other studies that reported that ApoA1 was positively correlated with vitamin D in Korean cohorts [37] and in Chinese osteoporosis patients [38]. In vitro studies have also indicated that vitamin D receptors regulate ApoA1 gene expression [39]. A meta-analysis of 7 RCTs demonstrated that daily vitamin D supplementation significantly increased ApoA1 levels [40], but no significant correlation between 25(OH) D and LDL-c was found in our study. In fact, the relationship between vitamin D and LDL-c is controversial according to previous studies. Chacko et al. observed no significant associations between serum 25(OH) D and LDL-c in the WHI-CaD population [41]. In contrast, however, a recent large cross-sectional study of 15,600 healthy patients provided evidence of a significant negative correlation between 25(OH) D and LDL-c levels [42]. These inconsistent results may be partly explained by the variations in the participant health status, age, BMI, and ethnicity.

Several observational studies have also shown that vitamin D deficiency is associated with elevated blood pressure, plasma glucose, and insulin resistance [8, 13, 43, 44]. However, our data revealed no consistent correlation of 25(OH) D levels with other cardiometabolic risk markers. In line with our study, numerous other studies have also failed to find significant associations between 25(OH) D levels and glucose metabolism or insulin sensitivity in both healthy populations and individuals with obesity [27, 45]. Further larger-scale prospective studies focusing on adults with severe obesity are warranted to confirm these findings.

The strength of this study lies in its contribution to the growing evidence regarding the relationship between vitamin D and cardiovascular risk factors in extremely obese populations. Our findings underscore the importance of vitamin D deficiency as a clinical correlate of CVD. However, this study has several limitations. First, the cross-sectional design precludes establishment of causal relationships between vitamin D levels and cardiovascular risk factors. Second, the number of participants with vitamin D sufficiency was low, so we had to combine vitamin D insufficiency and sufficiency into one group, restricting our capacity to examine associations at higher vitamin D concentrations. Third, the lack of data on physical activity, sunlight exposure, and dietary habits may have introduced confounding bias. Finally, we only enrolled Han Chinese subjects from North China, so our results may not be generalizable to other ethnic populations and geographic regions.

## 5. Conclusion

In this study, we identified a high prevalence of vitamin D deficiency among extremely obese individuals in Beijing, China. Serum 25(OH) D concentrations were inversely associated with the BMI and positively associated with HDL-c and ApoA1. Given the established links between the elevated BMI, dyslipidemia, and cardiometabolic morbidity, targeted strategies to address vitamin D deficiency in this population are clinically warranted. Furthermore, multicenter RCTs with large sample sizes are required to elucidate the potential impact of vitamin D supplementation on cardiovascular risk modulation.

## Figures and Tables

**Table 1:** Baseline clinical characteristics of participants stratified by sex.

	Total (*N* = 293)	Female (*N* = 222)	Male (*N* = 71)	*p* value
*N*	293	222 (75.8%)	71 (24.2%)	
Age (years)	36.61 ± 7.42	36.81 ± 7.49	35.99 ± 7.216	0.418
BMI (kg/m^2^)	42.04 ± 6.10	41.06 ± 4.94	45.13 ± 8.079	**< 0.001**
WC (cm)	120.00 (111.00, 132.00)	116.50 (109.75, 124.25)	133.00 (121.75, 144.25)	**< 0.001**
SBP (mmHg)	141.00 (129.00, 153.00)	140.00 (128.50, 151.50)	144.00 (131.25, 157.75)	0.104
DBP (mmHg)	84.00 (76.00, 94.00)	84.00 (76.00, 94.00)	86.00 (79.00, 94.00)	0.316
Season of 25(OH) D measurement				
Summer/fall (*N*, %)	170 (58%)	123 (55.4%)	47 (66.2%)	0.129
Winter/spring (*N*, %)	123 (42%)	99 (44.6%)	24 (33.8%)	
AST (U/L)	24.85 (18.35, 43.88)	24.45 (17.58, 43.63)	26.45 (20.95, 44.50)	**0.024**
ALT (U/L)	36.00 (22.90, 65.90)	33.70 (21.50, 65.80)	44.45 (27.25, 69.40)	0.188
Cre (μmol/L)	52.00 (44.85, 59.62)	49.60 (42.78, 54.65)	66.85 (56.45, 77.55)	**< 0.001**
UA (μmol/L)	421.00 (361.00, 503.75)	405.00 (349.00, 470.00)	500.00 (421.50, 572.50)	**< 0.001**
HbA1c (%)	6.79 ± 1.74	6.79 ± 1.81	6.79 ± 1.53	0.983
FBG (mmol/L)	6.98 ± 2.63	6.99 ± 2.70	6.92 ± 2.42	0.832
FINS (μIU/L)	27.18 (18.16, 36.96)	26.66 (18.17, 37.04)	29.23 (18.16, 37.08)	0.673
FCP (ng/mL)	2.82 (2.29, 3.49)	2.77 (2.19, 3.35)	3.18 (2.65, 3.98)	**0.001**
HOMA-IR	7.52 (4.97, 10.97)	7.28 (4.96, 11.19)	8.02 (5.69, 10.46)	0.631
LDL-c (mmol/L)	3.51 ± 0.93	3.57 ± 0.93	3.27 ± 0.94	**0.017**
HDL-c (mmol/L)	1.05 ± 0.23	1.08 ± 0.21	0.95 ± 0.25	**< 0.001**
ApoA1 (g/L)	1.28 ± 0.20	1.30 ± 0.19	1.20 ± 0.19	**0.001**
ApoB (g/L)	0.97 ± 0.21	0.97 ± 0.21	0.95 ± 0.21	0.427
PTH (pg/mL)	59.70 (45.71, 79.39)	62.35 (47.92, 80.70)	52.33 (40.68, 71.37)	**0.011**
Ca (mmol/L)	2.32 ± 0.10	2.32 ± 0.11	2.35 ± 0.31	0.185
P (mmol/L)	1.19 ± 0.23	1.18 ± 0.17	1.23 ± 0.34	0.103
Total 25(OH) D (ng/mL)	12.88 (9.94, 18.27)	12.52 (9.81, 16.99)	14.78 (10.91, 21.44)	**0.019**

*Note:* Continuous variables are expressed as mean ± SD or median (IQR) depending on data distribution. Categorical variables are shown as *n* (%). Differences in clinical characteristics between groups were examined by Student's *t*-test or the Mann–Whitney *U*-test for quantitative variables and the chi-squared test for categorical variables. *p* < 0.05 is highlighted in bold. ALT: alanine aminotransferase, AST: aspartate aminotransferase; Cre: creatinine; HbA1c: glycated hemoglobin; FINS: fasting plasma insulin; Ca: calcium; P: phosphorus; LDL-c: low-density lipoproteins; HDL-c: high-density lipoproteins; ApoA1: Apolipoprotein A1; ApoB: Apolipoprotein B.

Abbreviations: DBP, diastolic blood pressure; FBG, fasting blood glucose; FCP, fasting C-peptide; PTH, parathyroid hormone; SBP, systolic blood pressure; UA, uric acid; WC, waist circumference.

**Table 2:** Baseline clinical characteristics of participants stratified by 25(OH) D levels.

	Non–vitamin D deficiency	Vitamin D deficiency	*p* value
*N*	55 (18.8%)	238 (81.2%)	
Age (years)	36.12 ± 7.22	38.72 ± 7.97	**0.018**
BMI (kg/m^2^)	39.56 ± 4.15	42.62 ± 6.33	**0.001**
WC (cm)	116.00 (110.00, 128.00)	120.00 (112.00, 134.00)	0.177
SBP (mmHg)	144.00 (136.00, 154.00)	140.00 (128.00, 153.00)	0.153
DBP (mmHg)	87.00 (81.00, 96.00)	84.00 (75.00, 94.00)	0.159
Season of 25(OH) D measurement			0.289
Summer/fall (*N*, %)	28 (50.9%)	142 (59.7%)	
Winter/spring (*N*, %)	27 (49.1%)	96 (40.3%)	
AST (U/L)	24.00 (18.60, 41.70)	25.50 (18.25, 44.25)	0.823
ALT (U/L)	35.80 (22.50, 66.90)	36.00 (22.95, 65.85)	0.77
Cre (μmol/L)	53.20 (46.10, 65.40)	51.10 (44.70, 58.30)	**0.049**
UA (μmol/L)	420.00 (364.00, 550.00)	422.00 (359.00, 500.50)	0.441
HbA1c (%)	6.81 ± 1.82	6.79 ± 1.73	0.932
FBG (mmol/L)	7.07 ± 2.62	6.95 ± 2.64	0.77
FINS (μIU/L)	29.28 (18.02, 34.01)	26.74 (18.17, 37.68)	0.999
FCP (ng/mL)	2.80 (2.25, 3.61)	2.83 (2.29, 3.48)	0.965
HOMA-IR	8.11 (4.89, 11.60)	7.47 (4.98, 1.08)	0.855
LDL-c (mmol/L)	3.66 ± 0.99	3.47 ± 0.92	0.185
HDL-c (mmol/L)	1.11 ± 0.27	1.04 ± 0.22	**0.022**
ApoA1 (g/L)	1.34 ± 0.205	1.26 ± 0.193	**0.010**
ApoB (g/L)	1.02 ± 0.22	0.95 ± 0.21	0.086
PTH (pg/mL)	44.79 (38.88, 58.38)	63.73 (48.97, 82.53)	**< 0.001**
Ca (mmol/L)	2.35 ± 0.09	2.31 ± 0.11	**0.046**
P (mmol/L)	1.17 ± 0.15	1.20 ± 0.24	0.536

*Note:* Continuous variables are expressed as mean ± SD or median (IQR) depending on data distribution. Differences in clinical characteristics between groups were examined by Student's *t*-test or the Mann–Whitney *U*-test for quantitative variables and the chi-squared test for categorical variables. *p* < 0.05 is highlighted in bold. ALT: alanine aminotransferase; AST: aspartate aminotransferase; Cre: creatinine; HbA1c: glycated hemoglobin; FINS: fasting plasma insulin; LDL-c: low-density lipoproteins; HDL-c: high-density lipoproteins; ApoA1: Apolipoprotein A1; ApoB: Apolipoprotein B; Ca: calcium; P: phosphorus.

Abbreviations: DBP, diastolic blood pressure; FBG, fasting blood glucose; FCP, fasting C-peptide; PTH, parathyroid hormone; SBP, systolic blood pressure; UA, uric acid; WC, waist circumference.

**Table 3:** Multiple linear regression analysis: independent effect of circulating 25(OH) D on lipids.

Variables		25(OH) D		
		B	95% CI	*p* value
HDL-c	Crude	0.005	0.001 to 0.009	**0.014**
	Model 1	0.005	0.002 to 0.009	**0.006**
	Model 2	0.005	0.001 to 0.009	**0.007**

LDL-c	Crude	0.006	0.009 to 0.022	0.437
	Model 1	0.012	0.004 to 0.028	0.151
	Model 2	0.011	0.006 to 0.027	0.196

ApoA1	Crude	0.007	0.003 to 0.010	**< 0.001**
	Model 1	0.008	0.004 to 0.012	**< 0.001**
	Model 2	0.008	0.004 to 0.012	**< 0.001**

ApoB	Crude	0.001	0.003 to 0.005	0.487
	Model 1	0.002	0.002 to 0.006	0.347
	Model 2	0.002	0.003 to 0.006	0.463

*Note:* LDL-c: low-density lipoproteins; HDL-c: high-density lipoproteins; ApoA1: Apolipoprotein A1; ApoB: Apolipoprotein B. Model 1: adjusted for age, sex, and BMI. Model 2: adjusted for age, sex, BMI, and HOMA-IR. *p* < 0.05 is highlighted in bold.

## Data Availability

The data that support the findings of this study are available from the corresponding author upon reasonable request.

## References

[1] Collaborators (2017). Health Effects of Overweight and Obesity in 195 Countries Over 25 Years. *New England Journal of Medicine*.

[2] Pan X. F., Wang L., Pan A. (2021). Epidemiology and Determinants of Obesity in China. *Lancet Diabetes & Endocrinology*.

[3] Bjerregaard L. G., Jensen B. W., Angquist L., Osler M., Sorensen T. I. A., Baker J. L. (2018). Change in Overweight from Childhood to Early Adulthood and Risk of Type 2 Diabetes. *New England Journal of Medicine*.

[4] Arnold M., Leitzmann M., Freisling H. (2016). Obesity and Cancer: An Update of the Global Impact. *Cancer Epidemiology*.

[5] Twig G., Yaniv G., Levine H. (2016). Body-Mass Index in 2.3 Million Adolescents and Cardiovascular Death in Adulthood. *New England Journal of Medicine*.

[6] Bennour I., Haroun N., Sicard F., Mounien L., Landrier J. F. (2022). Vitamin D and Obesity/Adiposity-A Brief Overview of Recent Studies. *Nutrients*.

[7] Agarwal P., Agarwal Y., Hameed M. (2023). Recent Advances in Association between Vitamin D Levels and Cardiovascular Disorders. *Current Hypertension Reports*.

[8] Corica D., Zusi C., Olivieri F. (2019). Vitamin D Affects Insulin Sensitivity and Beta-Cell Function in Obese Non-diabetic Youths. *European Journal of Endocrinology*.

[9] Muscogiuri G., Sorice G. P., Ajjan R. (2012). Can Vitamin D Deficiency Cause Diabetes and Cardiovascular Diseases? Present Evidence and Future Perspectives. *Nutrition, Metabolism, and Cardiovascular Diseases*.

[10] Tang Z., Huang S., Ma R., Zheng H., Zhu Y. (2020). Low Vitamin D Status Is Associated with Obesity but No Other Cardiovascular Risk Factors in Chinese Children and Adolescents. *Nutrition, Metabolism, and Cardiovascular Diseases*.

[11] Barbarawi M., Kheiri B., Zayed Y. (2019). Vitamin D Supplementation and Cardiovascular Disease Risks in More Than 83 000 Individuals in 21 Randomized Clinical Trials: A Meta-Analysis. *JAMA Cardiology*.

[12] Holick M. F. (2009). Vitamin D Status: Measurement, Interpretation, and Clinical Application. *Annals of Epidemiology*.

[13] Lu L., Yu Z., Pan A. (2009). Plasma 25-hydroxyvitamin D Concentration and Metabolic Syndrome Among Middle-Aged and Elderly Chinese Individuals. *Diabetes Care*.

[14] Zhao J., Xia W., Nie M. (2011). The Levels of Bone Turnover Markers in Chinese Postmenopausal Women: Peking Vertebral Fracture Study. *Menopause*.

[15] Du X., Greenfield H., Fraser D. R., Ge K., Trube A., Wang Y. (2001). Vitamin D Deficiency and Associated Factors in Adolescent Girls in Beijing. *The American Journal of Clinical Nutrition*.

[16] Khodabakhshi A., Mahmoudabadi M., Vahid F. (2022). The Role of Serum 25 (OH) Vitamin D Level in the Correlation between Lipid Profile, Body Mass Index (BMI), and Blood Pressure. *Clinical Nutrition ESPEN*.

[17] Huang C. Y., Chang H. H., Lu C. W., Tseng F. Y., Lee L. T., Huang K. C. (2015). Vitamin D Status and Risk of Metabolic Syndrome Among Non-diabetic Young Adults. *Clinical Nutrition*.

[18] Lee J. H., Kim Y. A., Kim Y. S., Lee Y., Seo J. H. (2023). Association between Vitamin D Deficiency and Clinical Parameters in Men and Women Aged 50 Years or Older: A Cross-Sectional Cohort Study. *Nutrients*.

[19] Hyppoönen E., Power C. (2006). Vitamin D Status and Glucose Homeostasis in the 1958 British Birth Cohort: the Role of Obesity. *Diabetes Care*.

[20] Rafiq S., Jeppesen P. B. (2018). Body Mass Index, Vitamin D, and Type 2 Diabetes: A Systematic Review and Meta-Analysis. *Nutrients*.

[21] Zhang H. X., Zhai L., Gao Z., Yuan J. (2022). Relationship between Serum Vitamin D and Perirenal Fat Thickness in Patients with Metabolic Syndrome in Community. *Diabetes, Metabolic Syndrome and Obesity: Targets and Therapy*.

[22] Cheng S., Massaro J. M., Fox C. S. (2010). Adiposity, Cardiometabolic Risk, and Vitamin D Status: the Framingham Heart Study. *Diabetes*.

[23] Duan L., Han L., Liu Q., Zhao Y., Wang L., Wang Y. (2020). Effects of Vitamin D Supplementation on General and Central Obesity: Results from 20 Randomized Controlled Trials Involving Apparently Healthy Populations. *Annals of Nutrition & Metabolism*.

[24] Wortsman J., Matsuoka L. Y., Chen T. C., Lu Z., Holick M. F. (2000). Decreased Bioavailability of Vitamin D in Obesity. *The American Journal of Clinical Nutrition*.

[25] Drincic A. T., Armas L. A., Van Diest E. E., Heaney R. P. (2012). Volumetric Dilution, rather Than Sequestration Best Explains the Low Vitamin D Status of Obesity. *Obesity*.

[26] Florez H., Martinez R., Chacra W., Strickman-Stein N., Levis S. (2007). Outdoor Exercise Reduces the Risk of Hypovitaminosis D in the Obese. *The Journal of Steroid Biochemistry and Molecular Biology*.

[27] Tabesh M., Callegari E. T., Gorelik A. (2018). Associations between 25-hydroxyvitamin D Levels, Body Composition and Metabolic Profiles in Young Women. *European Journal of Clinical Nutrition*.

[28] Jorde R., Figenschau Y., Hutchinson M., Emaus N., Grimnes G. (2010). High Serum 25-hydroxyvitamin D Concentrations Are Associated with a Favorable Serum Lipid Profile. *European Journal of Clinical Nutrition*.

[29] Vitezova A., Zillikens M. C., van Herpt T. T. (2015). Vitamin D Status and Metabolic Syndrome in the Elderly: the Rotterdam Study. *European Journal of Endocrinology*.

[30] Huang X., Yang Y., Jiang Y., Zhou Z., Zhang J. (2023). Association between Vitamin D Deficiency and Lipid Profiles in Overweight and Obese Adults: a Systematic Review and Meta-Analysis. *BMC Public Health*.

[31] Kazlauskaite R., Powell L. H., Mandapakala C., Cursio J. F., Avery E. F., Calvin J. (2010). Vitamin D Is Associated with Atheroprotective High-Density Lipoprotein Profile in Postmenopausal Women. *Journal of Clinical Lipidology*.

[32] Mirhosseini N., Rainsbury J., Kimball S. M. (2018). Vitamin D Supplementation, Serum 25(OH)D Concentrations and Cardiovascular Disease Risk Factors: A Systematic Review and Meta-Analysis. *Frontiers in Cardiovascular Medicine*.

[33] Yang Y., Yan S., Yao N. (2023). Effects of Vitamin D Supplementation on the Regulation of Blood Lipid Levels in Prediabetic Subjects: A Meta-Analysis. *Frontiers in Nutrition*.

[34] Yu K. H., Song W. T., Tu X. Y., Zhou K., Prabahar K. (2025). The Effect of Vitamin D on the Lipid Profile in Individuals with Overweight or Obesity: A Meta-Analysis and Systematic Review of Randomized Controlled Trials. *Prostaglandins & Other Lipid Mediators*.

[35] Ponda M. P., Huang X., Odeh M. A., Breslow J. L., Kaufman H. W. (2012). Vitamin D May Not Improve Lipid Levels: a Serial Clinical Laboratory Data Study. *Circulation*.

[36] Su X., Peng D. Q. (2020). The Exchangeable Apolipoproteins in Lipid Metabolism and Obesity. *Clinica Chimica Acta*.

[37] Lee Y., Yoon J. W., Kim Y. A., Choi H. J., Yoon B. W., Seo J. H. (2022). A Genome-wide Association Study of Genetic Variants of Apolipoprotein A1 Levels and Their Association with Vitamin D in Korean Cohorts. *Genes*.

[38] Xu S. m, Lu K., Yang X. (2023). Association of 25-hydroxyvitamin D Levels with Lipid Profiles in Osteoporosis Patients: a Retrospective Cross-Sectional Study. *Journal of Orthopaedic Surgery and Research*.

[39] Wehmeier K. R., Mazza A., Hachem S. (2008). Differential Regulation of Apolipoprotein A-I Gene Expression by Vitamin D Receptor Modulators. *Biochimica et Biophysica Acta (BBA)-General Subjects*.

[40] Radkhah N., Shabbidar S., Zarezadeh M., Safaeiyan A., Barzegar A. (2021). Effects of Vitamin D Supplementation on Apolipoprotein A1 and B100 Levels in Adults: Systematic Review and Meta-Analysis of Controlled Clinical Trials. *Journal of Cardiovascular and Thoracic Research*.

[41] Chacko S. A., Song Y., Manson J. E. (2011). Serum 25-hydroxyvitamin D Concentrations in Relation to Cardiometabolic Risk Factors and Metabolic Syndrome in Postmenopausal Women. *The American Journal of Clinical Nutrition*.

[42] Gholamzad A., Khakpour N., Kabipour T., Gholamzad M. (2023). Association between Serum Vitamin D Levels and Lipid Profiles: a Cross-Sectional Analysis. *Scientific Reports*.

[43] Wolters M., Marron M., Foraita R. (2023). Longitudinal Associations between Vitamin D Status and Cardiometabolic Risk Markers Among Children and Adolescents. *Journal of Clinical Endocrinology and Metabolism*.

[44] Amirkhizi F., Pishdadian A., Asghari S., Hamedi-Shahraki S. (2021). Vitamin D Status Is Favorably Associated with the Cardiovascular Risk Factors in Adults with Obesity. *Clinical Nutrition ESPEN*.

[45] de las Heras J., Rajakumar K., Lee S., Bacha F., Holick M. F., Arslanian S. A. (2013). 25-Hydroxyvitamin D in Obese Youth across the Spectrum of Glucose Tolerance from Normal to Prediabetes to Type 2 Diabetes. *Diabetes Care*.

